# Multimodal deep learning for liver cancer applications: a scoping review

**DOI:** 10.3389/frai.2023.1247195

**Published:** 2023-10-27

**Authors:** Aisha Siam, Abdel Rahman Alsaify, Bushra Mohammad, Md. Rafiul Biswas, Hazrat Ali, Zubair Shah

**Affiliations:** College of Science and Engineering, Hamad Bin Khalifa University, Qatar Foundation, Doha, Qatar

**Keywords:** multimodal, deep learning, liver cancer, EHR, imaging modality

## Abstract

**Background:**

Hepatocellular carcinoma is a malignant neoplasm of the liver and a leading cause of cancer-related deaths worldwide. The multimodal data combines several modalities, such as medical images, clinical parameters, and electronic health record (EHR) reports, from diverse sources to accomplish the diagnosis of liver cancer. The introduction of deep learning models with multimodal data can enhance the diagnosis and improve physicians' decision-making for cancer patients.

**Objective:**

This scoping review explores the use of multimodal deep learning techniques (i.e., combining medical images and EHR data) in diagnosing and prognosis of hepatocellular carcinoma (HCC) and cholangiocarcinoma (CCA).

**Methodology:**

A comprehensive literature search was conducted in six databases along with forward and backward references list checking of the included studies. PRISMA (Preferred Reporting Items for Systematic Reviews and Meta-Analyses) extension for scoping review guidelines were followed for the study selection process. The data was extracted and synthesized from the included studies through thematic analysis.

**Results:**

Ten studies were included in this review. These studies utilized multimodal deep learning to predict and diagnose hepatocellular carcinoma (HCC), but no studies examined cholangiocarcinoma (CCA). Four imaging modalities (CT, MRI, WSI, and DSA) and 51 unique EHR records (clinical parameters and biomarkers) were used in these studies. The most frequently used medical imaging modalities were CT scans followed by MRI, whereas the most common EHR parameters used were age, gender, alpha-fetoprotein AFP, albumin, coagulation factors, and bilirubin. Ten unique deep-learning techniques were applied to both EHR modalities and imaging modalities for two main purposes, prediction and diagnosis.

**Conclusion:**

The use of multimodal data and deep learning techniques can help in the diagnosis and prediction of HCC. However, there is a limited number of works and available datasets for liver cancer, thus limiting the overall advancements of AI for liver cancer applications. Hence, more research should be undertaken to explore further the potential of multimodal deep learning in liver cancer applications.

## 1. Introduction

Hepatocellular cancer (HCC) and cholangiocarcinoma (CCA) are two types of liver cancer that are responsible for significant morbidity and mortality worldwide (Lee et al., 2011). The early detection and diagnosis of these cancers are essential for improving patient outcomes, as the survival rate decreases with the advancement of the disease (Asafo-Agyei and Samant, 2023). Accurate diagnosis and staging of cancer are crucial for improving patient survival and treatment outcomes. Hepatocellular carcinoma (HCC) and cholangiocarcinoma (CCA) are liver cancer types requiring precise diagnosis and staging. Traditionally, imaging techniques such as computed tomography (CT), magnetic resonance imaging (MRI), whole slide image (biopsy), and ultrasound (US) have been used as the standard of practice for diagnosing and staging HCC and CCA (Zhou et al., 2019) along with clinical findings, biological markers and blood test [liver function test, Alfa fetoprotein (AFP) and inflammation-based index (IBI)] (Asafo-Agyei and Samant, 2023). These modalities are analyzed by experts, including pathologists, oncologists, and gastroenterologists, and remain the gold standard for diagnosis confirmation.

Recently, there has been an increasing interest in using artificial intelligence (AI) in the medical field, including the cancer and oncology. With the digitization of healthcare records, AI modes can efficiently utilize patient data. Electronic Health Record (EHR) of patients comprises comprehensive information regarding their medical history, diagnoses, treatments, laboratory results, radiology images, genetic profiles, and more (Kohli and Tan, 2016). Harnessing the potential of this vast data deluge is a significant challenge but also holds tremendous promise for Medical AI techniques (Mohsen et al., 2022). AI techniques using machine learning and deep learning models have emerged as powerful tools for extracting valuable insights from massive EHRs and developing multimodal AI methods (Zhou et al., 2019). It can utilize multiple modalities of data concurrently, such as CT, MRI, and US, along with clinical findings, biological markers, and blood test results, including liver function tests, Alfa fetoprotein (AFP), and the inflammation-based index (IBI), and is able to provide a more comprehensive and accurate picture of the internal structure and function of the liver (Zhou et al., 2019).

A multimodal AI refers to an AI framework that integrates and processes information from multiple modalities or sources, such as text and images. This approach enables the AI-based system to learn and make predictions based on information extracted from different data types, allowing for a more comprehensive and holistic understanding of the underlying information (Audebert et al., 2020; Mohsen et al., 2022). Multimodal data for HCC provide the integration of multiple data sources such as blood test reports, CT, MRI, and liver biopsy, and enables the chances of higher diagnostic accuracy compared to single modality data. Similarly, the combination of different data types helps in building better models of potential risk stratification. Moreover, by combining multiple modalities, multimodal AI techniques enhance the extraction of meaningful features and make accurate predictions (Zhou et al., 2019). Deep learning, a subset of machine learning, involves artificial neural networks with multiple layers to learn hierarchical representations of data. In multimodal AI, deep learning models are designed to handle and process different data types simultaneously, capturing their inherent relationships and interactions (Zhou et al., 2019). A multimodal AI technique is useful in aiding clinicians in predicting various aspects related to HCC and CCA. It can assist in extracting mutually exclusive information from the data that can help in treatment outcome prediction, prognosis estimation, survival prediction, staging, and diagnosis. By leveraging diverse data sources, the multimodal AI technique provides with valuable insights for defining optimal treatment strategies and personalized patient management plans (Zhou et al., 2019).

Several studies have investigated the use of multimodal AI combining different data modalities for diagnosing HCC and CCA. However, there is a need for a scoping review to summarize and synthesize the current evidence on this topic. We are confident that this scoping review will give readers a thorough understanding of the developments made in multimodal AI combining imaging data and EHR for liver cancer applications. The reader will also get knowledge of how deep learning models might be created to align data from diverse modalities for distinct therapeutic tasks. Additionally, by highlighting the dearth of multimodal data resources for medical imaging and EHR for liver cancer applications, this review will encourage the research community to produce more multimodal medical data. Since we include studies on multimodal deep learning-based AI techniques, we use the terms multimodal deep learning and multimodal AI interchangeably in this review.

## 2. Methods

This scoping review focused extensively on the studies that used multimodal data and deep learning techniques to predict and diagnose HCC. There are several steps followed in conducting this review as below.

### 2.1. Search sources

A comprehensive literature search was conducted in PubMed, Scopus, Google Scholar, ACM, IEEEXplore, and CINAHL databases using relevant keywords. The PubMed database also covers Medline. The search was limited to studies published in the English language from January 2018 till August 15, 2023, to capture the most recent developments in multimodal deep learning-based AI methods using imaging and EHR data. Our search focused specifically on studies from 2018 due to the significant increase in HCC multimodal studies during that period. By limiting our analysis to studies from 2018, we aimed to capture the most up-to-date and relevant findings in this rapidly evolving area of research.

### 2.2. Search terms

The search terms used in this study were: ((“artificial intelligence”) OR (“deep learning”)) AND ((“multi-modal”) OR (“multimodal”) OR (“electronic health record”) OR (“image^*^”)) AND ((“liver cancer”) OR (“hepatocellular carcinoma”) OR (“bile duct cancer”) OR (“cholangiocarcinoma”)). Two Boolean operators were introduced, the OR operator to combine keywords within each category and the AND operator to merge keywords across all categories.

### 2.3. Study eligibility criteria

We included studies that combined multimodal data, i.e., imaging and EHR. The multimodal data combined imaging data such as MRI and CT scans with clinical parameters such as laboratory test results and vitals. We included studies that reported deep learning techniques such as convolutional neural networks (CNNs), transformers, or neural networks in their methods. The aim of this study was to identify the use of multimodal deep learning techniques in liver cancer application. So, we excluded studies that used only traditional machine-learning techniques. The types of included studies were peer-reviewed articles, dissertations, book chapters, and conference proceedings published from 2018 to August 2023. Only English language texts were included. We excluded studies that did not combine medical imaging and EHR data and also excluded studies that developed models for diseases other than liver cancer. We excluded systematic reviews, abstracts, studies that used languages other than English, and studies that were published before the year 2018. The study selection process was carried out by three authors independently. Conflicts among them were resolved through mutual discussions and through validation by all the authors.

### 2.4. Data extraction

The data were extracted from the included studies using a standardized form, including information on the study design, sample size, population characteristics, AI methods, interventions, and outcomes. It was used to ensure accurate and precise documentation of significant information for each study. The data extraction form is provided in Appendix A.

## 3. Results

### 3.1. Study selection results

**Figure 1:**
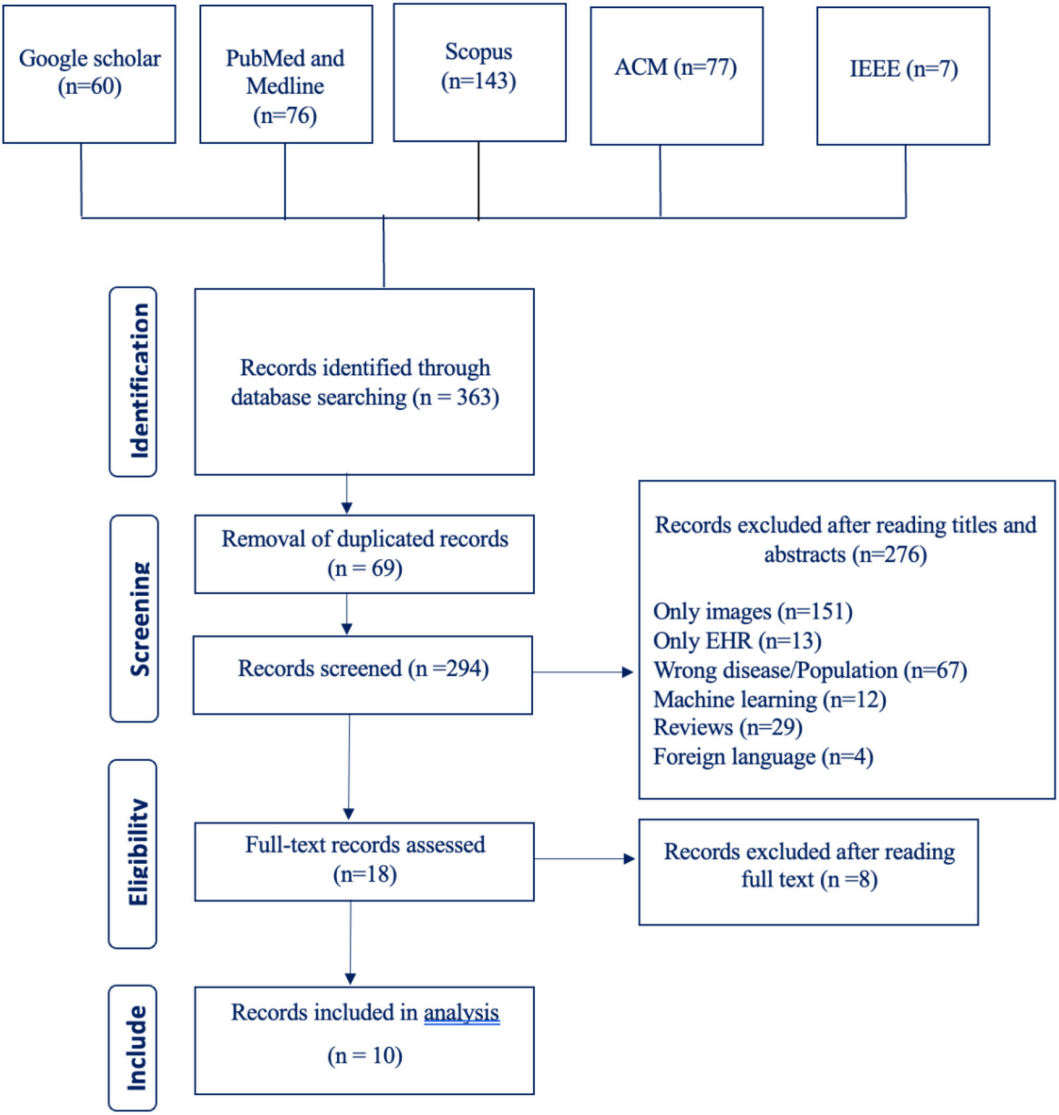
Prisma flow chart.

Our search terms yielded 363 studies from six different databases (Google Scholar 60, PubMed and Medline 76, Scopus 143, ACM 77, IEEE 7, CINAHL 0). After going through these studies' titles and abstracts, we excluded 276 studies and included 18 studies. The number of excluded studies with their reason for exclusion are listed in the PRISMA flowchart shown in Figure 1. After going through the full text of the studies, we excluded 8 studies and were left with a total of 10 studies.

Going through the demographic of the included 10 studies, we can see that 8 studies were from China and only 2 studies were from Brazil. During our literature review, we searched for studies published from 2018 to 2023; however, the final included studies were only published from 2020 to 2022. All the studies, except one conference paper, were journal articles.

### 3.2. Artificial intelligence techniques

The research encompassed multimodal AI techniques that are capable of identifying not only diagnostic parameters and biomarkers in EHRs, but also recognizing HCC abnormalities in medical image modalities. All of the studies focused on HCC, and no study involved CCA.

The purpose of employing multimodal deep learning models for HCC can be categorized into two main objectives: disease prediction and disease classification or diagnosis. Additionally, these models were utilized for predicting treatment response, determining survival rates, and staging the disease.

The primary deep learning architecture employed in these models was CNN. The multimodal deep learning models used a combination of two different deep learning neural network models trained on processing two different types of datasets modalities (medical imaging and EHRs). The included studies used models like VGG16, VGG19, Inception V3, and ResNet18, to extract and analyze detailed spatial features of medical images in creating multimodal AI techniques (Menegotto et al., 2020, 2021; Zhen et al., 2020; Gao et al., 2021; Hou et al., 2022; Zhang et al., 2022). Additionally, multi-task deep learning neural networks, UNet, MTNet, were used to integrate multiple modalities of data in addition to recurrent neural network (RNN) which can utilize, and process text information and numerical figures (clinical parameters and biological markers) derived from EHRs (Fu et al., 2021). Other techniques like Cox proportional hazards models' classifiers were used to develop predictive models from whole slides images (WSI) and clinical genetic data (Hou et al., 2022).

The studies included in this analysis employ multimodal deep-learning methods for the diagnosis and prediction of liver cancer. State-of-the-art deep learning models such as VGG19 and DeepAttnMISL, which is a state-of-the-art are employed for recognizing image modalities (Hou et al., 2022), while GhostNet/CNN, a combination of two single deep learning neural network models, is used for predicting treatment response to trans-arterial chemoembolization (TACE) (Sun et al., 2021).

Weighted gene co-expression network analysis (WGCNA) is used to analyze mRNA gene expression data from patients' files, while Cox-regression utilizes its outcome and the outcome from VGG16 (WSI processing model) to predict HCC patient survival (Hou et al., 2022). Multimodal AI techniques such as the multimodal Xception CNN and the Spatial Extractor-Temporal Encoder-Integration-Classifier (STIC) models are also used, which combine different modalities to improve diagnosis performance. Additionally, AI techniques combining deep learning architectures with machine learning methods such as SVM, Random Forest, and Cox regression are employed for survival analysis.

**Figure 2:**
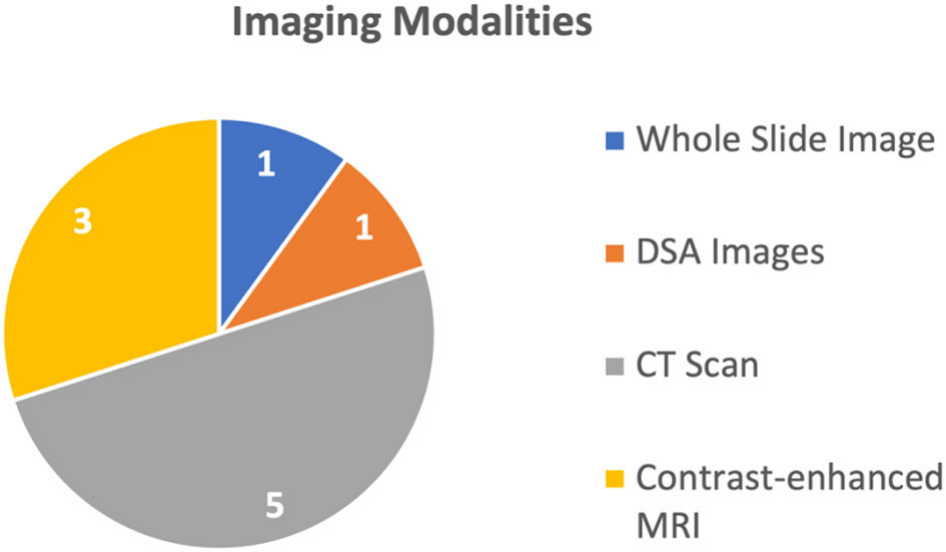
Image modalities used in the included studies.

Overall, these models and techniques have the potential to improve medical diagnosis, prediction, and survival analysis by integrating different data sources. By combining information from multiple modalities and utilizing advanced deep learning techniques, multimodal AI techniques can provide more accurate and reliable predictions, ultimately leading to improved patient outcomes. The 10 studies discussed four different types of medical images, explained in Figure 2. The most common imaging modality used in the studies was CT used in six studies (Liu et al., 2020; Menegotto et al., 2020, 2021; Fu et al., 2021; Gao et al., 2021; Sun et al., 2021), followed by MRI used in two studies (Zhen et al., 2020; Song et al., 2021), WSI used in one study (Hou et al., 2022), and Digital Subtraction Angiography (DSA) used in one study (Zhang et al., 2022), respectively. All these studies used single image modality and did not report combined use of multiple imaging modalities. Meanwhile, these multimodal deep learning models were capable of processing more than 1 biological marker or clinical parameter (in some models, the number of different types of clinical parameters was 22).

Prediction was the most common purpose for the use of multimodal AI techniques, addressed in six studies (Liu et al., 2020; Fu et al., 2021; Song et al., 2021; Sun et al., 2021; Hou et al., 2022; Zhang et al., 2022), while four studies (Menegotto et al., 2020, 2021; Zhen et al., 2020; Gao et al., 2021) used multimodal AI techniques for the purposes of diagnosis or classification of HCC (Menegotto et al., 2020, 2021; Zhen et al., 2020; Gao et al., 2021). The HCC predicting multimodal AI techniques are sub-categorized based on the type to survival prediction (Hou et al., 2022), Tran's catheter arterial chemoembolization (TACE) treatment response prediction (Sun et al., 2021), and microvascular invasion (MVI) prediction (Fu et al., 2021; Song et al., 2021). One study (Liu et al., 2020) introduced a multimodal AI model capable of performing all three types of predictions. For prediction purpose, the commonly used imaging modality was MRI and CT scans used in two studies (Song et al., 2021; Sun et al., 2021), followed by WSI and DSA, each used in one study (Hou et al., 2022; Zhang et al., 2022), respectively.

The remaining four studies used multimodal AI for diagnosis or classification of the HCC. Three of these studies used CT (Menegotto et al., 2020, 2021; Gao et al., 2021), while one study used MRI (Zhen et al., 2020). The summary of the usage of different imaging modalities is shown in Figure 2.

**Figure 3:**
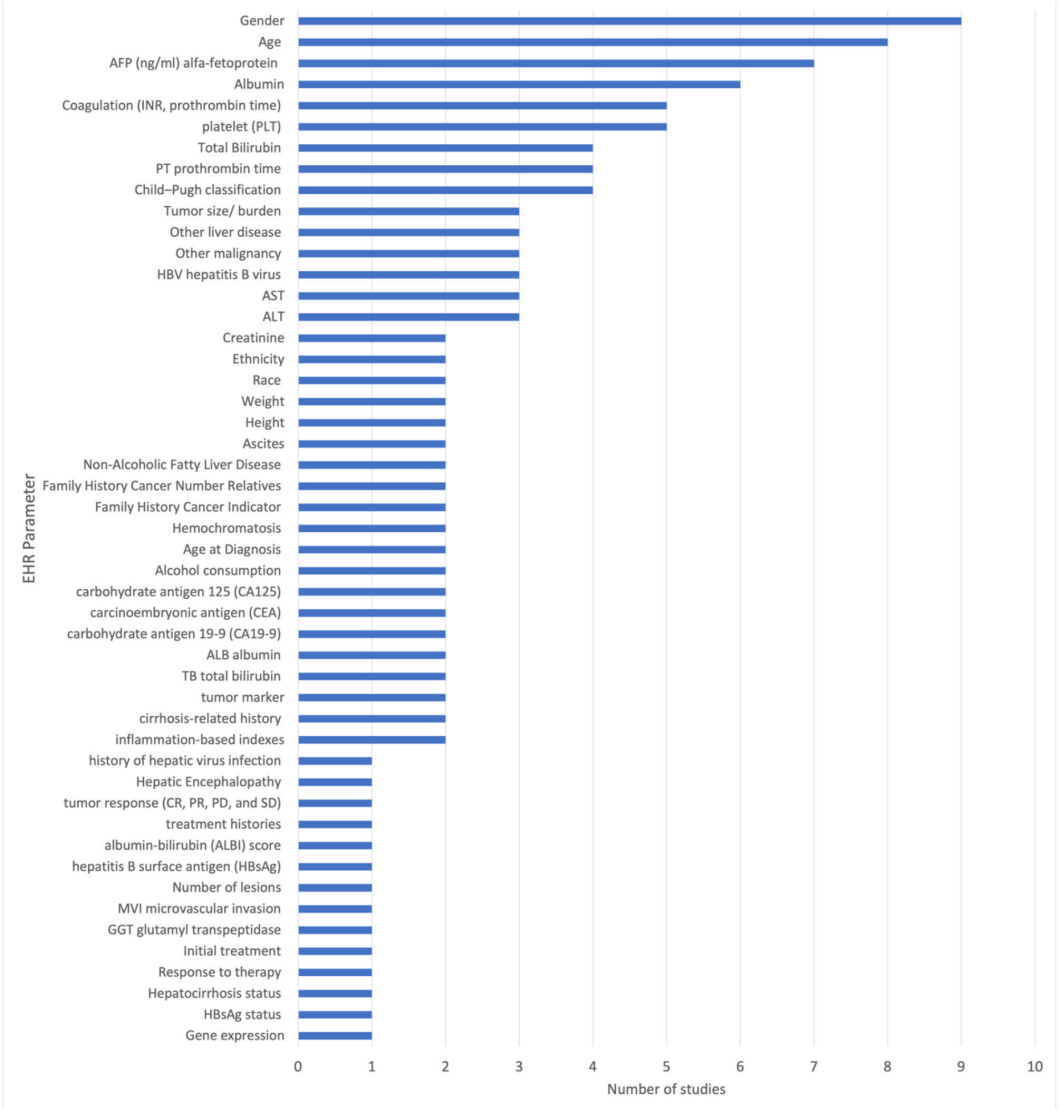
Summary of the number of studies using unique EHR parameters.

The included studies reported the use of 51 unique EHR parameters. Among the EHR related biomarkers and diagnostic parameters were patients age, gender, platelet (PLT), total bilirubin (TBIL), alpha fetoprotein (AFP), carbohydrate antigen 19-9 (CA19-9), carcinoembryonic antigen (CEA), carbohydrate antigen 125 (CA125), hepatitis B surface antigen (HBsAg), and liver function test. All other parameters are specified in Figure 3.

The most frequently used parameters are gender (reported in nine studies), age (reported in eight studies), alpha-fetoprotein (AFP) (reported in seven studies), platelet (PLT) count (reported in five studies), albumin (reported in six studies), and prothrombin time (PT) (reported in four studies). These parameters are used in multiple studies and are considered to be important in the diagnosis and prediction of liver cancer. Other commonly used parameters include total bilirubin (reported in four studies), Child-Pugh classification (reported in four studies), hepatitis B virus (HBV) (reported in three studies), ALT (reported in three studies), serum aspartate aminotransferase (AST) (reported in three studies), tumor marker (AFP, CEA, CA-125, CA19-9) (reported in two studies), carbohydrate antigen 19-9 (CA19-9) (reported in two studies), carcinoembryonic antigen (CEA) (reported in two studies), and carbohydrate antigen 125 (CA125) (reported in two studies).

Figure 3 shows the unique EHR parameters that various multimodal deep learning models can process. The Cox proportional hazards model is capable of processing 3/51 EHR modalities. GhostNet/CNN, a combination of GhostNet and convolutional neural network (CNN), can process 14 EHR modalities. Google Inception-ResNet V2 CNN, combined with an auto encoder neural network CNN, can process 16 EHR modalities. CNN+DLC, which combines CNN and deep learning classifier (DLC), can process 22 EHR modalities. Last but not least, the multi-task deep learning neural network (MTNet) is capable of processing 22 EHR parameters.

The model CNN combined with MTNet used the highest number of EHR parameters employing 22 clinical parameters (Song et al., 2021). Xception CNN used 20 clinical parameters (Menegotto et al., 2021), Google Inception-ResNet-V2 CNN used 16 clinical parameters (Zhen et al., 2020). GhostNet/CNN used 14 clinical parameters (Sun et al., 2021). Cox-Proportional Hazard (Cox-PH), CNN + Gated recurrent neural network (RNN) Spatial Extractor-Temporal Encoder-Integration-Classifier (STIC), and Cox proportional hazards model had the lowest number of EHR modalities, i.e., 9, 8, and 3, reported in Liu et al. (2020), Gao et al. (2021), and Hou et al. (2022), respectively. Table 1 summarizes each multimodal AI technique and the unique EHR parameters used to train the multi-modal deep learning model. Appendix explains the technical terms and the various names of deep learning models used in this text.

**Table 1:** Multimodal deep learning techniques.

**References**	**Deep learning models**	**Image modality used**	**EHR modality used (clinical parameters and biological markers)**	**Number of unique EHR parameters**	**Purposes of study**
Hou et al. (2022)	VGG19, Cox proportional hazards model (Cox-regression)	Whole slide images (WSI)	Gene expression (DCAF13, ELAC2, ZNF320, KIF18B, FERMT3)/gender and age.	3	Prediction: survival prediction
Sun et al. (2021)	GhostNet/CNN	MRI	Age, Gender, ALT, AST, HBsAg status, Child–Pugh classification, AFP (ng/ml), hepatocirrhosis status, response to therapy/inflammation-based indexes IBI (platelet, Lymphocyte, Monocite < neutrophil). Neutrophil-to-lymphocyte ratio (NLR), platelet-to-lymphocyte ratio (PLR), monocyte-to-lymphocyte ratio (MLR), systemic immune-inflammation index (SII), and neutrophil-to-lymphocyte ratio (SIRI) (clinical indexes are lymphocytes, platelets, monocytes, and neutrophils) (inflammatory indexes NLR, MLR, PLR, SII, and SIRI).	14	Prediction: treatment response (TACE)
Zhen et al. (2020)	Google Inception-ResNet V2 CNN + autoencoder neural network CNN	MRI	Clinical data was encoded using one-hot encoding. For example, gender. Age, gender, cirrhosis-related history, other cancers, tumor marker (AFP, CEA, CA-125, CA19-9, PSA, and Ferritin), and liver function (albumin, total bilirubin, prolonged prothrombin time, hepatic encephalopathy, and ascites).	16	Diagnosis: HCC
Song et al. (2021)	Radiomics, CNN	MRI	Age, gender, HBV hepatitis B virus, TB total bilirubin, ALB albumin, ALT alanine aminotransferase, GGT glutamyl transpeptidase, PT prothrombin time, AFP alpha fetoprotein, MVI microvascular invasion [The minimum Akaike information criterion (AIC) index was used as the stop criterion to determine the optimal characteristics. Then, the selected parameters were incorporated into the deep learning model to form the DLC model (Figure 1). Notably, for the selected parameters, categorical variables were encoded by one digit (i.e., −1 or 1 for each state), and continuous variables were normalized to (−0.5, 0.5)]. Neutrophils count, Lymphocytes count, INR, lob10AFP, and tumor size. Clinical parameters were collected, including sex, age, routine blood test, blood biochemical test, blood coagulation function test, markers of hepatic fibrosis, hepatitis virus B carriers, AFP, and tumor size. Serum component index, such as platelet-lymphocyte ratio (PLR), neutrophil-lymphocyte ratio (NLR), lymphocyte-to-monocyte ratio (LMR), prognostic nutritional index (PNI), aspartate aminotransferase-to-platelet ratio index (APRI), aspartate aminotransferase-to-neutrophil ratio index (ANRI) and aspartate aminotransferase-lymphocyte ratio (ALR), were calculated as previous reported.	22	Prediction: MVI (micro vascular invasion)
Fu et al. (2021)	UNet, radiomics, multi-task deep learning neural network (MTNet)	CT	Age, gender, initial treatment, HBV, Child-Pugh class, number of lesions, AFP level, Barcelona clinic liver cancer BCLC stages. Age, sex, Child-Pugh grade, HBV infection, and CT identified cirrhosis; tumor burden (location, lesion number, maximum diameters, alpha fetoprotein level, and BCLC stages); and initial treatments. We added nine qualitative radiological characteristics as previously reported: (22) fusion lesions, invasive shape, HCC capsule, HCC capsule breakthrough, corona enhancement, corona with low attenuation, mosaic architecture, nodule-in-nodule architecture, and enhancement ratio of the HCC lesions.	22	Prediction: MVI (micro vascular invasion)
Menegotto et al. (2020)	Deep convolutional neural network (DCNN)	CT	Anthropometric and sociodemographic: gender, age at diagnosis, height, weight, race E ethnicity—clinical: other malignancy, family history cancer indicator, family history cancer number relatives, alcohol consumption, hemochromatosis, hepatitis, non-alcoholic fatty liver disease, other liver disease—laboratory tests results: alpha-fetoprotein, platelets, prothrombin time, albumin, bilirubin, creatinine.	20	Diagnosis: HCC
Gao et al. (2021)	VGG16, Imagenet, CNN, Gated RNN	CT	Age, gender, platelet (PLT), total bilirubin (TBIL), alpha fetoprotein (AFP), carbohydrate antigen 19-9 (CA19-9), carcinoembryonic antigen (CEA), carbohydrate antigen 125 (CA125) and hepatitis B surface antigen (HBsAg).	8	Diagnosis: HCC
Menegotto et al. (2021)	Xception CNN	CT	Alpha-fetoprotein, bilirubin, platelets, weight, ethnicity, family history cancer number relatives, family history cancer indicator, other malignancy, race, gender, other liver disease, alcohol consumption, hepatitis, height, albumin, age at diagnosis, hemochromatosis, creatinine, prothrombin time, non-alcoholic fatty liver disease.	20	Diagnosis: HCC
Liu et al. (2020)	Cox-proportional hazard (Cox-PH)	CT	Age, sex, history of hepatic virus infection, Child-Pugh class, AFP, serum aspartate aminotransferase (AST), albumin-bilirubin (ALBI) score (39), treatment histories, and tumor response (CR, PR, PD, and SD).	9	Prediction: survival prediction
Zhang et al. (2022)	UNet model, ResNet, CNN	Digital subtraction angiography (DSA)	Clinical characteristics included age, sex, hepatitis B virus (HBV), a-Fetoprotein (AFP), prothrombin time (PT), and liver function parameters, which included Child–Pugh score, ascites, total bilirubin (TBIL), albumin (ALB), aspartate aminotransferase (AST), alanine aminotransferase (ALT), and C-reactive protein (CRP). All laboratory data were obtained within the 3 days before the first TACE session.	11	Prediction: treatment response (TACE)

#### 3.2.1. Implementation

The softwares used for the implementation of the multimodal deep learning models were Pytorch reported in three studies (Liu et al., 2020; Song et al., 2021; Hou et al., 2022) and TensorFlow reported in three studies (Zhen et al., 2020; Gao et al., 2021; Menegotto et al., 2021). One study also reported the use of LabelMe software tool (Zhang et al., 2022).

### 3.3. Datasets

#### 3.3.1. Data sources

The average number of samples for all the studies was 7,984, where the highest number was 38,424 MRI combined with 16 different clinical parameters per dataset, used in Zhen et al. (2020), and 37,084 CT scans combined with 20 clinical parameters per dataset, used in Menegotto et al. (2021). The size of the datasets of the remaining studies was between 145 and 766 with an average of 492 after removing the two extremes (Zhen et al., 2020; Menegotto et al., 2021). Four studies used datasets from open sources whereas the rest used datasets from private sources (Liu et al., 2020; Gao et al., 2021; Menegotto et al., 2021; Hou et al., 2022).

#### 3.3.2. Data sizes/training and testing

The training datasets were mentioned in all studies while validation and testing sets were not specified in some studies. Seven studies mentioned the validation set size (Menegotto et al., 2020, 2021; Zhen et al., 2020; Fu et al., 2021; Gao et al., 2021; Sun et al., 2021; Zhang et al., 2022) while only five studies mentioned the test set size (Menegotto et al., 2020, 2021; Gao et al., 2021; Song et al., 2021; Hou et al., 2022). The training time was mentioned in only five studies whereas it was not provided in the remaining studies. The highest epoch documented was 1,000, and the lowest was 20.

#### 3.3.3. Code availability

Only four studies provided links for the source code used for the development of multimodal deep learning models (Fu et al., 2021; Gao et al., 2021; Song et al., 2021; Hou et al., 2022). Table 2 summarizes the datasets categorization and how they were processed by the multimodal deep learning models (training, validation, and testing). The table also provides the data sources, training time, and type of modalities reported in each study.

**Table 2:** Description of datasets of liver cancer.

**Data sources (public or private)**	**Dataset size (number of samples)**				**Modality (type of images and the number of clinical parameters from EHR)**	**Number of epochs for training the model**	**References**
	**Training set**	**Validation set**	**Test set**	**Total**			
Public https://github.com/Houjiaxin123/Integrative-Histology-Genomic-HCC-Prognosis-Analysis	220	N/A	107	327	WSI + 3 clinical parameters	N/A	Hou et al., 2022
Private	319	80	N/A	399	MRI + 14 biological markers	50	Sun et al., 2021
Private	31,608	6816	N/A	38424	MRI + 16 biological markers	20	Zhen et al., 2020
Private	461	N/A	140	601	MRI + 22 clinical parameters	1,000	Song et al., 2021
Private	281	85	N/A	366	CT + 22 biological markers	N/A	Fu et al., 2021
Private	536	153	77	766	CT + 20 biological markers	N/A	Menegotto et al., 2020
Public https://github.com/ruitian-olivia/STIC-model	499	111	113	723	CT + 8 clinical parameters	50	Gao et al., 2021
Public https://github.com/amenegotto/pyLiver/blob/master/csv/clinical_data.csv	29,104	3,816	4,164	37,084	CT+ 20 clinical parameters	500	Menegotto et al., 2021
Public https://github.com/havakv/pycox/	145	N/A	N/A	145	CT + 9 clinical parameters	N/A	Liu et al., 2020
Private	360	245	N/A	605	DSA + 11 clinical parameters	N/A	Zhang et al., 2022

N/A, not available.

### 3.4. Validation/evaluation metrics

#### 3.4.1. Type of validation

Various metrics and validation techniques were used to evaluate the performance of the multimodal AI models. Six studies have mentioned the validation type namely, three studies reported 5-fold cross-validation (Zhen et al., 2020; Gao et al., 2021; Hou et al., 2022), two studies reported external validation (Fu et al., 2021; Gao et al., 2021) and one study reported 10-fold cross-validation (Menegotto et al., 2021). One study used both external validations along with 5-fold cross-validation (Gao et al., 2021).

#### 3.4.2. Evaluation metrics

**Figure 4:**
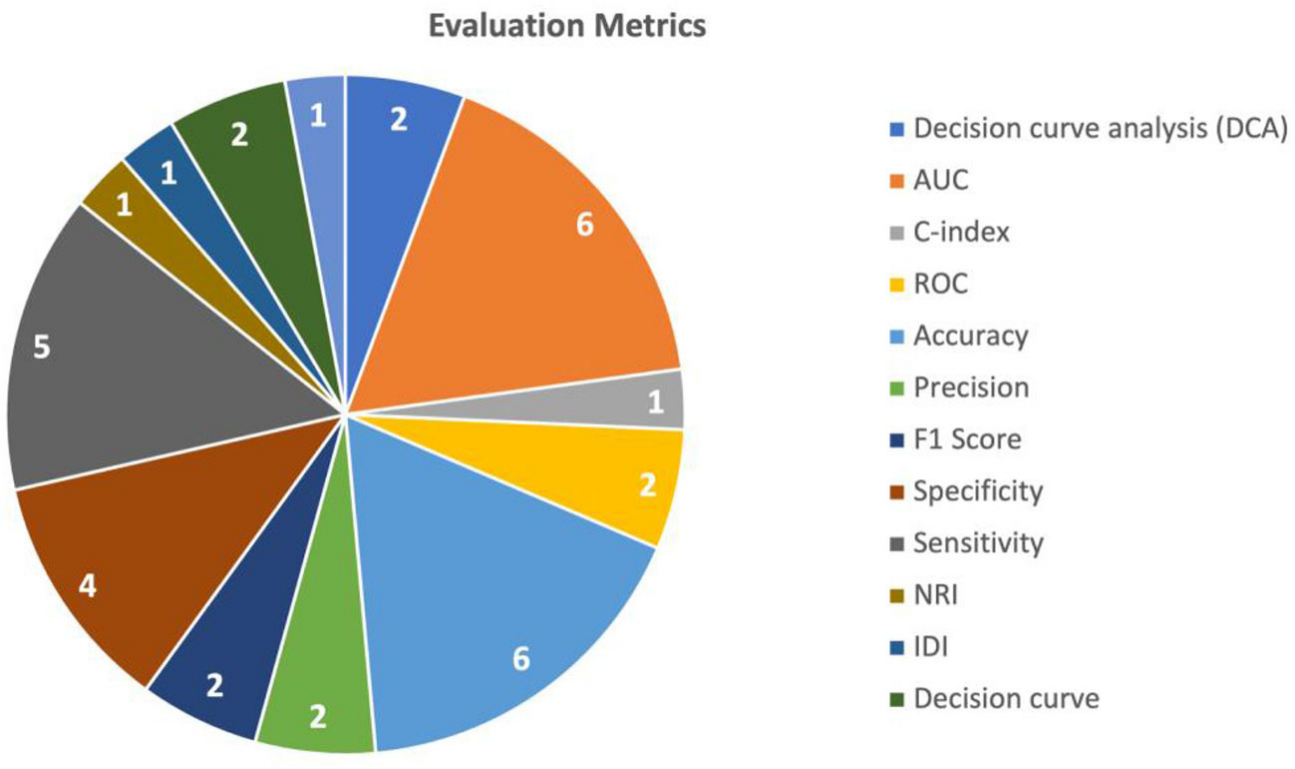
Evaluation metrics used in the studies.

To evaluate the performance of the multimodal AI models, various metrics and validation techniques were used for this purpose. The two most commonly utilized evaluation metrics in the included studies were AUC (Area under the Curve) and accuracy. AUC was used in seven studies (Liu et al., 2020; Zhen et al., 2020; Fu et al., 2021; Gao et al., 2021; Song et al., 2021; Sun et al., 2021; Hou et al., 2022) and accuracy was used in six studies (Zhen et al., 2020; Gao et al., 2021; Menegotto et al., 2021; Song et al., 2021; Sun et al., 2021; Zhang et al., 2022). Sensitivity was used in five studies (Zhen et al., 2020; Fu et al., 2021; Gao et al., 2021; Song et al., 2021; Zhang et al., 2022), and specificity was reported in five (Zhen et al., 2020; Fu et al., 2021; Gao et al., 2021; Song et al., 2021; Zhang et al., 2022). The performance of predicting HCC was mostly measured using the AUC (reported in five studies) (Liu et al., 2020; Fu et al., 2021; Song et al., 2021; Sun et al., 2021; Hou et al., 2022), followed by accuracy (Song et al., 2021; Sun et al., 2021; Zhang et al., 2022) and sensitivity (Fu et al., 2021; Song et al., 2021; Zhang et al., 2022) (each reported in three studies). The performance of HCC diagnosis was mainly tested using accuracy (reported in three studies) (Zhen et al., 2020; Gao et al., 2021; Menegotto et al., 2021), followed by AUC, sensitivity, and specificity (each reported in two studies) (Zhen et al., 2020; Gao et al., 2021). A summary of the commonly used metrics is shown in Figure 4.

Decision curve analysis (DCA) is a method used to evaluate the clinical value of the AI models. It involves comparing the net benefit of using the model to make clinical decisions with the net benefit of using a different decision-making strategy. Additionally, the use of various statistical tests like NRI (Net Reclassification Index), Integrated Discrimination Improvement (IDI), and calibration helped assess the models' performance. The use of different evaluation metrics is summarized in Table 3. Table 3 shows the minimum to maximum performance reported in the included studies for each evaluation metric.

**Table 3:** Summary of evaluation metrics used in included studies.

**Evaluation metrics**	**Performance result (%)**	**Number of studies**	**References**
Decision curve analysis	N/A	2	Fu et al., 2021; Hou et al., 2022
AUC	0.72–0.99	7	Liu et al., 2020; Zhen et al., 2020; Fu et al., 2021; Gao et al., 2021; Song et al., 2021; Sun et al., 2021; Hou et al., 2022
C-index	0.746	1	Hou et al., 2022
ROC	N/A	2	Fu et al., 2021; Hou et al., 2022
Accuracy	0.72–0.98	6	Zhen et al., 2020; Gao et al., 2021; Menegotto et al., 2021; Song et al., 2021; Hou et al., 2022; Zhang et al., 2022
Precision	0.89–0.97	2	Menegotto et al., 2021; Hou et al., 2022
F1 Score	0.86–0.98	2	Menegotto et al., 2021; Sun et al., 2021
Specificity	0.78–0.83	4	Zhen et al., 2020; Gao et al., 2021; Song et al., 2021; Zhang et al., 2022
Sensitivity	0.50–0.89	5	Zhen et al., 2020; Fu et al., 2021; Gao et al., 2021; Song et al., 2021; Zhang et al., 2022
NRI	N/A	1	Fu et al., 2021
IDI	N/A	1	Fu et al., 2021
Decision curve	N/A	2	Fu et al., 2021; Hou et al., 2022
Recall	0.75–0.86	1	Menegotto et al., 2021

Range of values show performance from minimum to maximum as reported in the included studies. N/A, not available.

## 4. Discussion

### 4.1. Research implications

There are certain challenges related to the multimodal deep learning models developed in the included studies (Sun et al., 2021; Hou et al., 2022). Firstly, the models lack enough multi-centers data, which hinders the ability to evaluate its performance effectively. This insufficiency in diverse and representative data sets reduces the reliability and generalizability of the model's results. Secondly, the potential relationship between different modalities within the multimodal deep learning model is not adequately understood or clearly defined. The model's ability to accurately represent complex relationships and phenomena is compromised without a comprehensive understanding of how these modalities interact and influence each other. Furthermore, the research results primarily focus on the application level, meaning that they predominantly address practical uses rather than investigating the underlying mechanisms responsible for the observed outcomes. This limitation restricts the depth of understanding achieved by the study and leaves gaps in the comprehension of the fundamental processes involved (Hou et al., 2022).

To address these shortcomings, several suggestions are reported. Firstly, exploring effective algorithms that can extract relevant and meaningful information from multimodal data is recommended. This step is crucial for improving the model's performance and enhancing its ability to leverage the diverse information contained within different modalities. Secondly, this study emphasizes the need to model the connections between modalities. Researchers can improve the model's accuracy and predictive capabilities by establishing clear and comprehensive models that capture the relationships and interactions between different modalities. Lastly, the study proposes the use of computational representations grounded in biological discoveries. Biological insights and principles can enhance the model's validity and align it more closely with natural systems' underlying mechanisms and processes. Addressing these challenges and implementing the suggested solutions would strengthen the multimodal deep learning model, enhancing its reliability, explanatory power, and potential for advancing scientific understanding in the field.

Three of the included studies were conducted retrospectively, meaning that they analyzed past data and events to draw conclusions (Liu et al., 2020; Zhen et al., 2020; Sun et al., 2021). To ensure the applicability of the findings across the entire range of liver diseases encountered in clinical practice, future training should include a larger number of patients with specific types of focal liver diseases. To broaden the scope of the research, it would be ideal to include less common liver masses in future studies. Examples of such masses could be abscesses, adenomas, and rare malignancies. By incorporating these less frequent liver masses, a more comprehensive understanding of the diverse spectrum of liver diseases can be achieved, leading to improved diagnostic and treatment approaches. Furthermore, conducting high-quality prospective studies involving multiple medical centers is crucial. These studies should be designed to gather data in real time, allowing for more accurate and up-to-date assessments of the effectiveness and outcomes of different diagnostic and treatment approaches. This is particularly important for high-risk patients with cirrhosis, as their specific needs and challenges warrant specialized attention and investigation. By incorporating these recommendations, future research efforts can enhance the breadth and depth of knowledge in the field of liver diseases, enabling more precise and effective management strategies for patients across the full range of liver pathologies encountered in clinical practice.

Two studies acknowledge that the deep learning architectures are often perceived as a “black box” due to their complex and intricate nature (Fu et al., 2021; Song et al., 2021). This means that the inner workings of a deep learning model and the specific correlation between the features used in the model and TS results are not easily explainable or interpretable. Despite demonstrating the stability of their final deep learning model, the study recognizes the limitation of not being able to provide a pathological interpretation for deep learning radiomics. This refers to the inability to directly relate the outputs or predictions of the deep learning models to specific pathological changes observed in cases of HCC. The “black box” effect commonly encountered in deep learning studies implies that the model's decision-making process and the reasons behind its predictions are not transparent or easily understood. Therefore, it becomes challenging to establish a clear connection between the features used in the deep learning model and the pathological changes that occur in HCC.

To address this challenge, further research is needed to explore and establish the relationship between the deep learning model's predictions and the actual pathological changes observed in HCC. This indicates the need to delve deeper into understanding how the deep learning model's outputs align with the underlying pathological mechanisms and processes associated with the disease. By conducting additional research and investigations, researchers should shed light on the “black box” nature of the deep learning method, elucidate the correlation between relevant features used in the model and the outcomes, and ultimately provide a clearer pathological interpretation of the deep learning radiomics in the context of HCC.

The studies utilized different deep learning models for processing medical images and electronic health record (EHR) data separately and then combined the results with building the multimodal AI model. There was no single variant of CNN architecture used across all the studies. CT scans were the most commonly used medical imaging modality, followed by MRI. We identified more than 50 clinical parameters and biomarkers related to HCC that were used to train and test the multimodal AI models. However, none of the studies included the history of jaundice or bile duct disorders as part of the clinical parameters, despite their importance as signs of liver abnormalities.

It is worth noting that the number of EHR modalities processed does not necessarily correspond to the performance of the deep learning model. Other factors, such as the model architecture and training data also play important roles in determining the model's performance.

The multimodal AI techniques lacked multi-centers data and the potential relationship between modalities was not clear. To address these challenges, effective algorithms for extracting multimodal data information should be explored, computational representations based on biological discoveries should be used, larger populations and multicenter studies should be conducted, and feature selection techniques and clinical indexes should be employed.

However, this scoping review allows researchers to investigate more uses of multimodal AI for the diagnosis of HCC and to study multimodal AI techniques developed for recognizing more medical imaging modalities. It also encourages researchers to study multimodal AI techniques for purposes other than those mentioned in this paper and to start conducting similar studies in CCA. Datasets utilized by the included studies are mainly from private sources and are used as training and validation datasets. Some studies do not mention the type of validation adopted for the model training and evaluation.

### 4.2. Limitations

While our scoping review offers valuable insights into the use of multimodal AI in liver cancer research, it is important to acknowledge the limitations of our study. One major limitation is that some of the datasets used in the studies were not fully described, leaving questions about their labeling and generalizability. Additionally, the lack of specificity regarding data sources used in the studies could make it difficult to reproduce or compare the models' performances. Finally, we focused on studies published within the past 5 years, which may have limited the scope of our analysis but captured the most recent development in the field of multimodal deep learning for the detection and diagnosis of liver cancer. The specific inclusion criteria on EHR+images data also limited the number of studies.

Despite these limitations, our review provides a foundation for understanding the application of Multimodal AI techniques in liver cancer research. By identifying common variables and models used across studies, we can better assess the potential of these models in improving HCC prediction and treatment response.

## 5. Conclusion

The detection and prognosis of liver cancer, or HCC, have recently been facilitated by recent advancements in deep learning-based AI techniques. In this scoping review, we analyzed 10 studies that investigated the application of multimodal deep learning models in HCC. We did not find any studies related to the use of multimodal deep learning models for CCA. The studies focused primarily on HCC prediction rather than HCC classification or diagnosis, with a particular emphasis on predicting response to TACE treatment. Overall, the studies highlighted the potential of multimodal AI for improving HCC prediction and treatment response assessment, but more research is needed to explore their effectiveness in other areas of liver cancer research. Multimodal AI techniques have the capacity to simultaneously evaluate vast quantities of complex data, including medical images and electronic health records, and infer useful patterns and insights. With the use of this technology, HCC diagnoses might be made more accurately, and the course of the disease could be predicted, which may ultimately lead to better outcomes and a higher survival rate. However, these models face limitations such as the lack of diverse data sets, unclear relationships between modalities, and a focus on explanations and understanding of the underlying mechanisms. Suggestions include exploring effective algorithms, establishing clear inter-modality relationships, and incorporating biological insights. In the context of HCC, the studies reviewed in this work primarily focused on HCC prediction and treatment response assessment using different multimodal AI techniques. However, limitations such as the “black box” nature of deep learning and the need for pathological interpretations persist. Future research should address these limitations, expand to other liver diseases, and incorporate larger populations and multicenter studies for comprehensive understanding and improved diagnostic and treatment approaches.

## Data availability statement

The original contributions presented in the study are included in the article/Supplementary material, further inquiries can be directed to the corresponding authors.

## Author contributions

AS, AA, and BM performed a search in different databases and were involved in the study inclusion, exclusion, and data extraction. MB resolved if there were any conflicts among AS, AA, and BM and reviewed the scoping review process. HA and ZS conceptualized the study and guided this scoping review. All authors agreed on the final draft of the manuscript.
